# Potassium 2-[(2-carboxy­phen­yl)disulfan­yl]benzoate–2,2′-disulfanediyl­dibenzoic acid (1/1)

**DOI:** 10.1107/S1600536809049824

**Published:** 2009-11-25

**Authors:** Hadi D. Arman, Pavel Poplaukhin, Edward R. T. Tiekink

**Affiliations:** a Department of Chemistry, The University of Texas at San Antonio, One UTSA Circle, San Antonio, Texas 78249-0698, USA; b Chemical Abstracts Service, 2540 Olentangy River Rd, Columbus, Ohio 43202, USA; c Department of Chemistry, University of Malaya, 50603 Kuala Lumpur, Malaysia

## Abstract

In the title compound, K^+^·C_14_H_9_O_4_S_2_^−^·C_14_H_10_O_4_S_2_, the hydrogen 2,2′-dithio­dibenzoate and 2,2′-disulfane­diyl­di­ben­zoic acid species combine to provide an O_6_S_2_ donor set to the potassium cation based on a cubic geometry. K⋯S [3.1733 (7) and 3.5499 (8) Å] and K⋯O [2.6586 (16)–3.0661 (15) Å)] inter­actions, coupled with O—H⋯O hydrogen bonding, lead to the formation of supra­molecular chains along [010].

## Related literature

For terminology of co-crystals, see: Zukerman-Schpector & Tiekink (2008 ▶). For related studies on co-crystal formation with 2,2′-disulfanediyl­dibenzoic acid, see: Broker & Tiekink (2007 ▶); Broker *et al.* (2008 ▶).
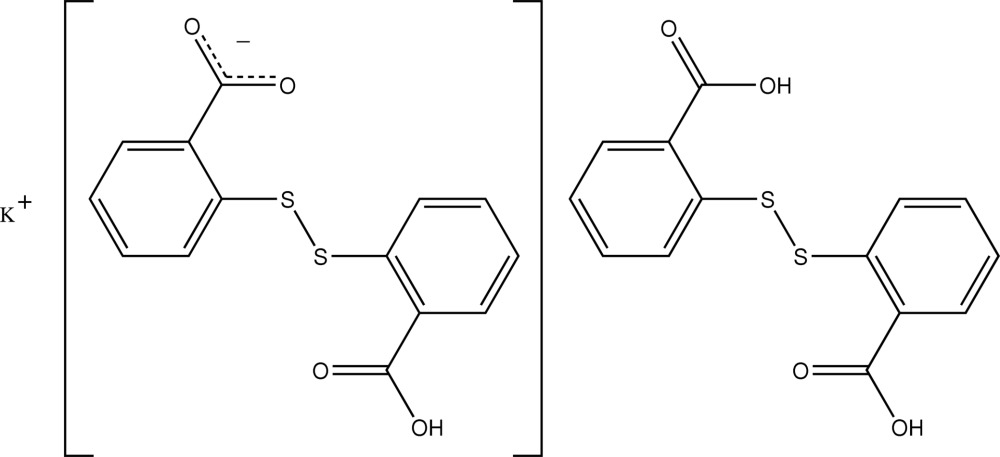


## Experimental

### 

#### Crystal data

K^+^·C_14_H_9_O_4_S_2_^−^·C_14_H_10_O_4_S_2_
*M* *_r_* = 650.81
Triclinic, 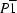

*a* = 11.1128 (14) Å
*b* = 12.1225 (15) Å
*c* = 12.5344 (12) Å
α = 65.501 (8)°
β = 64.216 (8)°
γ = 80.144 (10)°
*V* = 1383.5 (3) Å^3^
*Z* = 2
Mo *K*α radiation
μ = 0.55 mm^−1^
*T* = 98 K
0.50 × 0.25 × 0.20 mm

#### Data collection

Rigaku AFC12K/SATURN724 diffractometer
Absorption correction: multi-scan (*ABSCOR*; Higashi, 1995 ▶) *T*_min_ = 0.840, *T*_max_ = 1
10251 measured reflections
6302 independent reflections
5938 reflections with *I* > 2σ(*I*)
*R*_int_ = 0.030

#### Refinement

*R*[*F* ^2^ > 2σ(*F* ^2^)] = 0.041
*wR*(*F* ^2^) = 0.102
*S* = 1.07
6302 reflections
379 parameters
3 restraints
H-atom parameters constrained
Δρ_max_ = 0.39 e Å^−3^
Δρ_min_ = −0.43 e Å^−3^

### 

Data collection: *CrystalClear* (Rigaku/MSC, 2005 ▶); cell refinement: *CrystalClear*; data reduction: *CrystalClear*; program(s) used to solve structure: *SHELXS97* (Sheldrick, 2008 ▶); program(s) used to refine structure: *SHELXL97* (Sheldrick, 2008 ▶); molecular graphics: *DIAMOND* (Brandenburg, 2006 ▶); software used to prepare material for publication: *publCIF* (Westrip, 2009 ▶).

## Supplementary Material

Crystal structure: contains datablocks global, I. DOI: 10.1107/S1600536809049824/hg2606sup1.cif

Structure factors: contains datablocks I. DOI: 10.1107/S1600536809049824/hg2606Isup2.hkl

Additional supplementary materials:  crystallographic information; 3D view; checkCIF report

## Figures and Tables

**Table 1:** Hydrogen-bond geometry (Å, °)

*D*—H⋯*A*	*D*—H	H⋯*A*	*D*⋯*A*	*D*—H⋯*A*
O4—H1o⋯O1^i^	0.84	1.80	2.631 (2)	169
O6—H2o⋯O2^ii^	0.84	1.68	2.515 (2)	177
O8—H3o⋯O5^i^	0.84	1.88	2.704 (2)	167

Symmetry codes: (i) 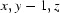; (ii) 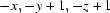.

**Fig. 1.**
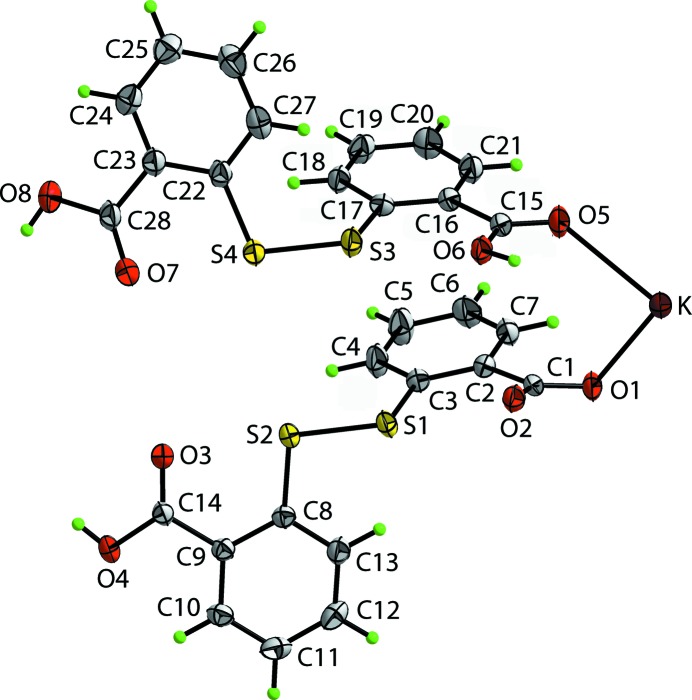
Molecular structures of the asymmetric unit of (I), showing atom-labelling scheme and displacement ellipsoids at the 50% probability level.

**Fig. 2.**
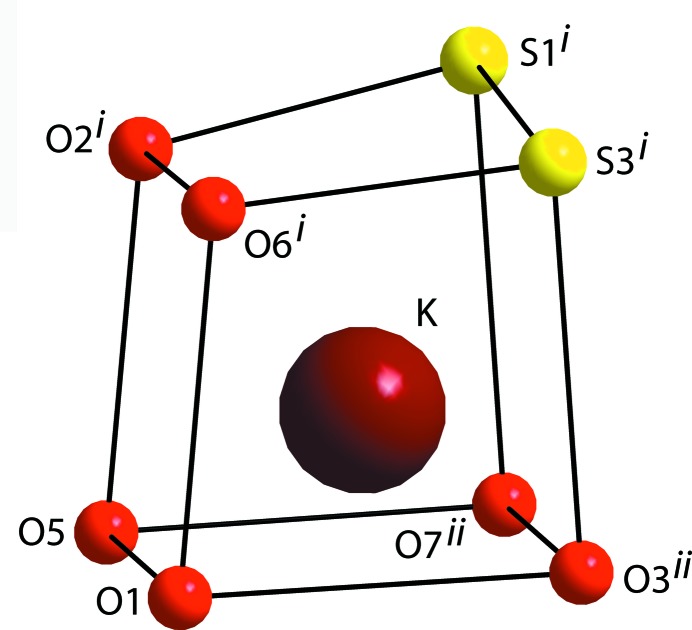
The coordination environment for the K+ cation in (I), highlighting the approximately cubic geometry defined by an O6S2 donor set. Symmetry codes: i, -x, 1 - y, 1 - z; ii, x, 1 + y, z. Colour code: K, brown; S, yellow; and O, red.

**Fig. 3.**
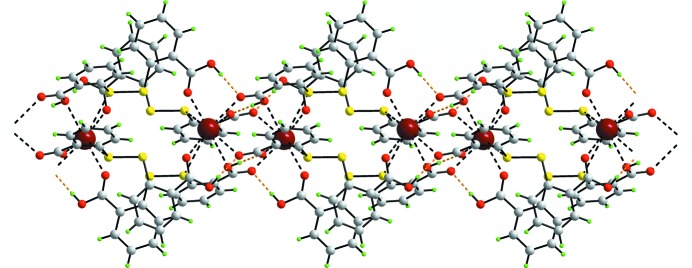
Supramolecular chain in (I) oriented along [0 1 0]. Colour code: K, brown; S, yellow; O,red; N, blue; C, grey; and H, green. The O–H···O hydrogen bonds are shown as orange dashed lines.

## supplementary crystallographic information

### Comment

The title compound, (I), a co-crystal of the potassium salt of hydrogen 2,2'-disulfanediyldibenzoate with 2,2'-disulfanediyldibenzoic acid (Zukerman-Schpector & Tiekink, 2008) was isolated during on-going studies into co-crystal formation of 2,2'-disulfanediyldibenzoic acid (Broker & Tiekink, 2007; Broker *et al.*, 2008). The asymmetric unit, Fig. 1, comprises a potassium cation, a hydrogen 2,2'-disulfanediyldibenzoate anion and a neutral 2,2'-disulfanediyldibenzoic acid molecule. Confirmation of deprotonation of the C1-carboxylate is seen in the C1–O1 and C1–O2 bond distances of 1.251 (2) and 1.273 (2) Å, respectively, with their near equivalence contrasting the disparity in the C–O bond distances for the C14-, C15- and C-28 carboxylic acid groups where the *C*–O_carbonyl_ bonds (1.215 (2) to 1.234 (2) Å) are systematically shorter than the *C*–O_hydroxyl_ bonds (1.299 (2) to 1.328 (2) Å). The respective carboxylic acid groups are co-planar with the benzene rings to which they are bonded. By contrast, the C1-carboxylate group is somewhat twisted out of the plane as seen in the O1–C1–C2–C3 torsion angle of 160.18 (18) °. Both the hydrogen 2,2'-disulfanediyldibenzoate and 2,2'-disulfanediyldibenzoic acid species adopt the common *L*-conformation (Broker & Tiekink, 2007) as seen in the dihedral angles formed between the (C2–C7) and (C8–C13) rings of 78.65 (10) °, and between the (C16–C21) and (C22–C27) rings of 75.41 (11) °.

The K^+^ cation geometry is defined by six O atoms (range of K···O = 2.6586 (16) to 3.0661 (15) Å) and two S atoms (K···S = 3.1733 (7) and 3.5499 (8) Å). The coordination geometry is based on a cube, Fig. 2, with one face defined by the O1, O5, O3^ii^, and O7^ii^ atoms, and the other by the S1^i^, S3^1^, O2^i^, and O7^i^ atoms; see the caption to Fig. 2 for symmetry operations. The dihedral angle formed between the two approximately square faces is 8.95 (4) °.

The K^+^ function as bridges between the 2,2'-disulfanediyldibenzoate and 2,2'-disulfanediyldibenzoic acid species to generate a supramolecular chain aligned along [0 1 0], Fig. 3. Stability to the chain is afforded by O–H···O hydrogen bonds, Table 1. The supramolecular chains are consolidated into the crystal structure by contacts of the type C–H···π: C25–H···*Cg*(C2–C7)^iii^ = 2.57 Å, C25···*Cg*(C2–C7)^iii^ = 3.456 (2) Å with an angle at H25 = 155 °; symmetry operation iii: *x*, *y*, -1 + *z*.

### Experimental

2,2'-disulfanediyldibenzoic acid (306 mg, 1.00 mmol) and 85% potassium hydroxide (132 mg, 2.00 mmol) were dissolved in methanol (40 ml). The resulting cloudy solution was filtered and left to evaporate slowly. Colourless block-like crystals of (I) appeared in five days.

### Refinement

The C-bound H-atoms were placed in calculated positions (C–H 0.95 Å) and were included in the refinement in the riding model approximation with *U*_iso_(H) set to 1.2*U*_eq_(C). The O-bound H-atoms were located in a difference Fourier map and refined with an O–H restraint of 0.840±0.001 Å, and with *U*_iso_(H) = 1.5*U*_eq_(O).

### Crystal data

**Table:**

K^+^·C_14_H_9_O_4_S_2_^−^·C_14_H_10_O_4_S_2_	*Z* = 2
*M**_r_* = 650.81	*F*(000) = 668
Triclinic, *P*1	*D*_x_ = 1.562 Mg m^−^^3^
Hall symbol: -P 1	Mo *K*α radiation, λ = 0.71069 Å
*a* = 11.1128 (14) Å	Cell parameters from 5714 reflections
*b* = 12.1225 (15) Å	θ = 2.7–40.6°
*c* = 12.5344 (12) Å	µ = 0.55 mm^−^^1^
α = 65.501 (8)°	*T* = 98 K
β = 64.216 (8)°	Prism, colourless
γ = 80.144 (10)°	0.50 × 0.25 × 0.20 mm
*V* = 1383.5 (3) Å^3^	

### Data collection

**Table:**

Rigaku AFC12K/SATURN724 diffractometer	6302 independent reflections
Radiation source: fine-focus sealed tube	5938 reflections with *I* > 2σ(*I*)
graphite	*R*_int_ = 0.030
ω scans	θ_max_ = 27.5°, θ_min_ = 2.3°
Absorption correction: multi-scan (*ABSCOR*; Higashi, 1995)	*h* = −14→14
*T*_min_ = 0.840, *T*_max_ = 1	*k* = −12→15
10251 measured reflections	*l* = −16→16

### Refinement

**Table:**

Refinement on *F*^2^	Primary atom site location: structure-invariant direct methods
Least-squares matrix: full	Secondary atom site location: difference Fourier map
*R*[*F*^2^ > 2σ(*F*^2^)] = 0.041	Hydrogen site location: inferred from neighbouring sites
*wR*(*F*^2^) = 0.102	H-atom parameters constrained
*S* = 1.07	*w* = 1/[σ^2^(*F*_o_^2^) + (0.0443*P*)^2^ + 0.8893*P*] where *P* = (*F*_o_^2^ + 2*F*_c_^2^)/3
6302 reflections	(Δ/σ)_max_ = 0.001
379 parameters	Δρ_max_ = 0.39 e Å^−^^3^
3 restraints	Δρ_min_ = −0.43 e Å^−^^3^

### Special details

**Table:**

Geometry. All e.s.d.'s (except the e.s.d. in the dihedral angle between two l.s. planes) are estimated using the full covariance matrix. The cell e.s.d.'s are taken into account individually in the estimation of e.s.d.'s in distances, angles and torsion angles; correlations between e.s.d.'s in cell parameters are only used when they are defined by crystal symmetry. An approximate (isotropic) treatment of cell e.s.d.'s is used for estimating e.s.d.'s involving l.s. planes.
Refinement. Refinement of *F*^2^ against ALL reflections. The weighted *R*-factor *wR* and goodness of fit *S* are based on *F*^2^, conventional *R*-factors *R* are based on *F*, with *F* set to zero for negative *F*^2^. The threshold expression of *F*^2^ > σ(*F*^2^) is used only for calculating *R*-factors(gt) *etc*. and is not relevant to the choice of reflections for refinement. *R*-factors based on *F*^2^ are statistically about twice as large as those based on *F*, and *R*- factors based on ALL data will be even larger.

### Fractional atomic coordinates and isotropic or equivalent isotropic displacement parameters (Å^2^)

**Table:**

	*x*	*y*	*z*	*U*_iso_*/*U*_eq_	
K	0.10124 (4)	0.69989 (3)	0.47189 (4)	0.01869 (10)	
S1	0.11089 (5)	0.14205 (4)	0.63434 (5)	0.01768 (11)	
S2	0.14595 (5)	−0.02859 (4)	0.63245 (4)	0.01697 (11)	
S3	0.18089 (5)	0.27372 (4)	0.26012 (5)	0.02144 (11)	
S4	0.21527 (5)	0.10430 (4)	0.25588 (5)	0.02251 (12)	
O1	0.18078 (14)	0.49091 (12)	0.62398 (14)	0.0211 (3)	
O2	0.05044 (13)	0.37852 (12)	0.61416 (13)	0.0185 (3)	
O3	0.15180 (14)	−0.25033 (11)	0.63792 (13)	0.0189 (3)	
O4	0.10686 (16)	−0.42661 (12)	0.80702 (14)	0.0246 (3)	
H1O	0.1205	−0.4501	0.7484	0.037*	
O5	0.26289 (15)	0.60519 (12)	0.28654 (15)	0.0243 (3)	
O6	0.12413 (14)	0.47773 (12)	0.30945 (13)	0.0195 (3)	
H2O	0.0681	0.5271	0.3349	0.029*	
O7	0.24704 (17)	−0.12001 (13)	0.26137 (15)	0.0295 (3)	
O8	0.35737 (16)	−0.17734 (13)	0.09558 (15)	0.0264 (3)	
H3O	0.3404	−0.2465	0.1547	0.040*	
C1	0.16289 (19)	0.40409 (15)	0.60292 (17)	0.0155 (3)	
C2	0.28063 (18)	0.32770 (16)	0.56010 (17)	0.0160 (3)	
C3	0.27134 (19)	0.20991 (16)	0.56926 (18)	0.0173 (3)	
C4	0.3891 (2)	0.14730 (18)	0.5273 (2)	0.0252 (4)	
H4	0.3841	0.0671	0.5341	0.030*	
C5	0.5130 (2)	0.20089 (19)	0.4760 (2)	0.0294 (5)	
H5	0.5920	0.1574	0.4472	0.035*	
C6	0.5225 (2)	0.31767 (19)	0.4663 (2)	0.0269 (4)	
H6	0.6073	0.3545	0.4313	0.032*	
C7	0.4064 (2)	0.37927 (17)	0.50859 (19)	0.0212 (4)	
H7	0.4124	0.4591	0.5023	0.025*	
C8	0.12319 (18)	−0.12228 (16)	0.79411 (17)	0.0161 (3)	
C9	0.10333 (18)	−0.24877 (16)	0.84089 (17)	0.0157 (3)	
C10	0.07086 (19)	−0.31939 (17)	0.97067 (18)	0.0196 (4)	
H10	0.0549	−0.4039	1.0022	0.023*	
C11	0.0616 (2)	−0.26811 (18)	1.05393 (19)	0.0226 (4)	
H11	0.0371	−0.3163	1.1425	0.027*	
C12	0.0886 (2)	−0.14551 (18)	1.00653 (19)	0.0224 (4)	
H12	0.0863	−0.1105	1.0626	0.027*	
C13	0.11874 (19)	−0.07327 (17)	0.87901 (19)	0.0200 (4)	
H13	0.1367	0.0107	0.8486	0.024*	
C14	0.12207 (18)	−0.30683 (16)	0.75195 (18)	0.0164 (3)	
C15	0.24360 (19)	0.51477 (16)	0.27414 (17)	0.0172 (3)	
C16	0.35667 (19)	0.44214 (16)	0.21775 (17)	0.0169 (3)	
C17	0.34242 (19)	0.33352 (16)	0.20805 (18)	0.0176 (3)	
C18	0.4573 (2)	0.27481 (17)	0.15355 (19)	0.0204 (4)	
H18	0.4492	0.2016	0.1465	0.024*	
C19	0.5836 (2)	0.32104 (18)	0.1093 (2)	0.0228 (4)	
H19	0.6607	0.2793	0.0727	0.027*	
C20	0.5976 (2)	0.42820 (19)	0.1182 (2)	0.0247 (4)	
H20	0.6839	0.4605	0.0872	0.030*	
C21	0.4851 (2)	0.48689 (17)	0.1724 (2)	0.0219 (4)	
H21	0.4947	0.5598	0.1792	0.026*	
C22	0.26830 (19)	0.12903 (17)	0.09047 (19)	0.0196 (4)	
C23	0.31293 (19)	0.03064 (17)	0.05098 (19)	0.0201 (4)	
C24	0.3623 (2)	0.05123 (19)	−0.0790 (2)	0.0243 (4)	
H24	0.3940	−0.0151	−0.1055	0.029*	
C25	0.3657 (2)	0.1666 (2)	−0.1700 (2)	0.0275 (4)	
H25	0.4004	0.1799	−0.2583	0.033*	
C26	0.3180 (2)	0.26238 (19)	−0.1302 (2)	0.0281 (4)	
H26	0.3172	0.3415	−0.1916	0.034*	
C27	0.2713 (2)	0.24465 (18)	−0.0023 (2)	0.0240 (4)	
H27	0.2410	0.3119	0.0226	0.029*	
C28	0.3029 (2)	−0.09482 (17)	0.1464 (2)	0.0212 (4)	

### Atomic displacement parameters (Å^2^)

**Table:**

	*U*^11^	*U*^22^	*U*^33^	*U*^12^	*U*^13^	*U*^23^
K	0.0236 (2)	0.01461 (18)	0.0195 (2)	0.00072 (15)	−0.00917 (16)	−0.00773 (15)
S1	0.0179 (2)	0.0111 (2)	0.0223 (2)	0.00063 (16)	−0.00674 (18)	−0.00661 (17)
S2	0.0223 (2)	0.0113 (2)	0.0166 (2)	−0.00094 (16)	−0.00695 (18)	−0.00533 (16)
S3	0.0165 (2)	0.0181 (2)	0.0269 (3)	−0.00257 (17)	−0.00043 (19)	−0.01389 (19)
S4	0.0238 (2)	0.0162 (2)	0.0217 (2)	−0.00477 (18)	0.00022 (19)	−0.01007 (18)
O1	0.0246 (7)	0.0139 (6)	0.0254 (7)	0.0003 (5)	−0.0072 (6)	−0.0112 (5)
O2	0.0186 (6)	0.0169 (6)	0.0212 (7)	0.0016 (5)	−0.0065 (5)	−0.0104 (5)
O3	0.0248 (7)	0.0149 (6)	0.0165 (6)	−0.0021 (5)	−0.0063 (6)	−0.0068 (5)
O4	0.0392 (9)	0.0120 (6)	0.0212 (7)	−0.0032 (6)	−0.0102 (7)	−0.0058 (5)
O5	0.0239 (7)	0.0180 (6)	0.0305 (8)	−0.0013 (6)	−0.0049 (6)	−0.0148 (6)
O6	0.0183 (6)	0.0157 (6)	0.0236 (7)	0.0018 (5)	−0.0049 (6)	−0.0111 (5)
O7	0.0394 (9)	0.0165 (7)	0.0240 (8)	−0.0050 (6)	−0.0025 (7)	−0.0086 (6)
O8	0.0331 (8)	0.0183 (7)	0.0275 (8)	0.0024 (6)	−0.0093 (7)	−0.0125 (6)
C1	0.0210 (9)	0.0098 (7)	0.0126 (8)	−0.0003 (6)	−0.0047 (7)	−0.0035 (6)
C2	0.0185 (8)	0.0140 (8)	0.0144 (8)	−0.0004 (7)	−0.0046 (7)	−0.0064 (7)
C3	0.0180 (8)	0.0149 (8)	0.0171 (9)	−0.0009 (7)	−0.0045 (7)	−0.0067 (7)
C4	0.0208 (10)	0.0187 (9)	0.0338 (11)	0.0018 (7)	−0.0044 (8)	−0.0157 (8)
C5	0.0188 (10)	0.0246 (10)	0.0410 (13)	0.0030 (8)	−0.0038 (9)	−0.0192 (9)
C6	0.0184 (9)	0.0237 (10)	0.0316 (11)	−0.0032 (8)	−0.0023 (8)	−0.0114 (9)
C7	0.0225 (9)	0.0145 (8)	0.0233 (10)	−0.0014 (7)	−0.0054 (8)	−0.0078 (7)
C8	0.0157 (8)	0.0148 (8)	0.0154 (8)	−0.0003 (6)	−0.0044 (7)	−0.0057 (7)
C9	0.0152 (8)	0.0145 (8)	0.0158 (8)	−0.0008 (6)	−0.0049 (7)	−0.0056 (7)
C10	0.0185 (9)	0.0179 (8)	0.0164 (9)	−0.0034 (7)	−0.0034 (7)	−0.0037 (7)
C11	0.0211 (9)	0.0268 (10)	0.0142 (9)	0.0014 (8)	−0.0040 (7)	−0.0064 (7)
C12	0.0234 (9)	0.0273 (10)	0.0215 (10)	0.0064 (8)	−0.0097 (8)	−0.0157 (8)
C13	0.0213 (9)	0.0178 (8)	0.0222 (9)	0.0018 (7)	−0.0075 (8)	−0.0109 (7)
C14	0.0164 (8)	0.0126 (8)	0.0182 (9)	−0.0009 (6)	−0.0046 (7)	−0.0062 (7)
C15	0.0220 (9)	0.0125 (8)	0.0147 (8)	0.0006 (7)	−0.0056 (7)	−0.0050 (6)
C16	0.0197 (9)	0.0140 (8)	0.0149 (8)	0.0007 (7)	−0.0054 (7)	−0.0054 (7)
C17	0.0175 (8)	0.0167 (8)	0.0157 (8)	−0.0025 (7)	−0.0034 (7)	−0.0062 (7)
C18	0.0203 (9)	0.0175 (8)	0.0224 (9)	−0.0001 (7)	−0.0043 (8)	−0.0112 (7)
C19	0.0170 (9)	0.0250 (10)	0.0250 (10)	0.0032 (8)	−0.0043 (8)	−0.0139 (8)
C20	0.0175 (9)	0.0264 (10)	0.0303 (11)	−0.0014 (8)	−0.0073 (8)	−0.0130 (8)
C21	0.0225 (9)	0.0188 (9)	0.0256 (10)	−0.0018 (7)	−0.0081 (8)	−0.0105 (8)
C22	0.0168 (8)	0.0176 (8)	0.0232 (9)	−0.0028 (7)	−0.0037 (7)	−0.0102 (7)
C23	0.0197 (9)	0.0175 (9)	0.0235 (10)	−0.0011 (7)	−0.0064 (8)	−0.0102 (7)
C24	0.0266 (10)	0.0251 (10)	0.0265 (10)	0.0004 (8)	−0.0112 (9)	−0.0143 (8)
C25	0.0314 (11)	0.0294 (10)	0.0245 (10)	−0.0015 (9)	−0.0125 (9)	−0.0110 (9)
C26	0.0333 (11)	0.0213 (9)	0.0319 (11)	−0.0007 (8)	−0.0184 (10)	−0.0063 (8)
C27	0.0240 (10)	0.0176 (9)	0.0314 (11)	0.0005 (7)	−0.0112 (9)	−0.0105 (8)
C28	0.0228 (9)	0.0169 (9)	0.0262 (10)	−0.0030 (7)	−0.0079 (8)	−0.0113 (8)

### Geometric parameters (Å, °)

**Table:**

K—O7^i^	2.6586 (16)	C5—H5	0.9500
K—O3^i^	2.6806 (14)	C6—C7	1.381 (3)
K—O1	2.7436 (15)	C6—H6	0.9500
K—O5	2.7960 (15)	C7—H7	0.9500
K—O2^ii^	2.8086 (14)	C8—C13	1.399 (2)
K—O6^ii^	3.0661 (15)	C8—C9	1.415 (2)
K—S1^ii^	3.1733 (7)	C9—C10	1.396 (3)
K—S3^ii^	3.5499 (8)	C9—C14	1.477 (2)
S1—C3	1.7893 (19)	C10—C11	1.382 (3)
S1—S2	2.0465 (7)	C10—H10	0.9500
S1—K^ii^	3.1733 (7)	C11—C12	1.384 (3)
S2—C8	1.7899 (19)	C11—H11	0.9500
S3—C17	1.7910 (19)	C12—C13	1.381 (3)
S3—S4	2.0423 (7)	C12—H12	0.9500
S3—K^ii^	3.5499 (8)	C13—H13	0.9500
S4—C22	1.794 (2)	C15—C16	1.489 (2)
O1—C1	1.251 (2)	C16—C21	1.399 (3)
O2—C1	1.273 (2)	C16—C17	1.412 (2)
O2—K^ii^	2.8086 (14)	C17—C18	1.392 (3)
O3—C14	1.215 (2)	C18—C19	1.387 (3)
O3—K^iii^	2.6806 (14)	C18—H18	0.9500
O4—C14	1.328 (2)	C19—C20	1.389 (3)
O4—H1O	0.8400	C19—H19	0.9500
O5—C15	1.234 (2)	C20—C21	1.374 (3)
O6—C15	1.299 (2)	C20—H20	0.9500
O6—K^ii^	3.0661 (15)	C21—H21	0.9500
O6—H2O	0.8401	C22—C27	1.396 (3)
O7—C28	1.216 (3)	C22—C23	1.410 (3)
O7—K^iii^	2.6586 (16)	C23—C24	1.397 (3)
O8—C28	1.326 (2)	C23—C28	1.480 (3)
O8—H3O	0.8400	C24—C25	1.384 (3)
C1—C2	1.498 (2)	C24—H24	0.9500
C2—C7	1.393 (3)	C25—C26	1.385 (3)
C2—C3	1.402 (2)	C25—H25	0.9500
C3—C4	1.399 (3)	C26—C27	1.383 (3)
C4—C5	1.386 (3)	C26—H26	0.9500
C4—H4	0.9500	C27—H27	0.9500
C5—C6	1.389 (3)		
			
O7^i^—K—O3^i^	96.77 (5)	C6—C5—H5	119.8
O7^i^—K—O1	129.86 (5)	C7—C6—C5	118.79 (19)
O3^i^—K—O1	71.45 (4)	C7—C6—H6	120.6
O7^i^—K—O5	71.64 (5)	C5—C6—H6	120.6
O3^i^—K—O5	131.40 (5)	C6—C7—C2	121.89 (17)
O1—K—O5	80.67 (4)	C6—C7—H7	119.1
O7^i^—K—O2^ii^	102.50 (5)	C2—C7—H7	119.1
O3^i^—K—O2^ii^	156.62 (4)	C13—C8—C9	118.12 (17)
O1—K—O2^ii^	104.90 (4)	C13—C8—S2	121.69 (14)
O5—K—O2^ii^	68.35 (4)	C9—C8—S2	120.13 (14)
O7^i^—K—O6^ii^	165.18 (5)	C10—C9—C8	119.83 (17)
O3^i^—K—O6^ii^	91.66 (4)	C10—C9—C14	119.97 (16)
O1—K—O6^ii^	64.50 (4)	C8—C9—C14	120.16 (16)
O5—K—O6^ii^	111.36 (4)	C11—C10—C9	120.93 (18)
O2^ii^—K—O6^ii^	66.83 (4)	C11—C10—H10	119.5
O7^i^—K—S1^ii^	76.34 (4)	C9—C10—H10	119.5
O3^i^—K—S1^ii^	119.10 (3)	C10—C11—C12	119.14 (18)
O1—K—S1^ii^	152.42 (3)	C10—C11—H11	120.4
O5—K—S1^ii^	104.08 (4)	C12—C11—H11	120.4
O2^ii^—K—S1^ii^	54.55 (3)	C13—C12—C11	121.05 (18)
O6^ii^—K—S1^ii^	88.92 (3)	C13—C12—H12	119.5
O7^i^—K—C1	132.30 (5)	C11—C12—H12	119.5
O3^i^—K—C1	91.89 (4)	C12—C13—C8	120.76 (18)
O1—K—C1	20.67 (4)	C12—C13—H13	119.6
O5—K—C1	67.73 (4)	C8—C13—H13	119.6
O2^ii^—K—C1	84.91 (4)	O3—C14—O4	122.96 (17)
O6^ii^—K—C1	59.17 (4)	O3—C14—C9	122.95 (16)
S1^ii^—K—C1	137.02 (4)	O4—C14—C9	114.07 (16)
O7^i^—K—S3^ii^	126.93 (4)	O5—C15—O6	122.01 (17)
O3^i^—K—S3^ii^	63.62 (3)	O5—C15—C16	121.54 (17)
O1—K—S3^ii^	92.08 (3)	O6—C15—C16	116.45 (15)
O5—K—S3^ii^	158.01 (3)	C21—C16—C17	119.09 (17)
O2^ii^—K—S3^ii^	93.93 (3)	C21—C16—C15	116.14 (16)
O6^ii^—K—S3^ii^	47.48 (3)	C17—C16—C15	124.77 (17)
S1^ii^—K—S3^ii^	73.298 (18)	C18—C17—C16	118.50 (17)
C1—K—S3^ii^	98.84 (4)	C18—C17—S3	120.19 (14)
C3—S1—S2	106.32 (6)	C16—C17—S3	121.29 (14)
C3—S1—K^ii^	117.10 (6)	C19—C18—C17	121.31 (17)
S2—S1—K^ii^	117.18 (2)	C19—C18—H18	119.3
C8—S2—S1	103.19 (6)	C17—C18—H18	119.3
C17—S3—S4	105.25 (6)	C18—C19—C20	120.18 (18)
C17—S3—K^ii^	122.79 (6)	C18—C19—H19	119.9
S4—S3—K^ii^	115.73 (3)	C20—C19—H19	119.9
C22—S4—S3	104.75 (7)	C21—C20—C19	119.20 (19)
C1—O1—K	108.58 (11)	C21—C20—H20	120.4
C1—O2—K^ii^	150.37 (12)	C19—C20—H20	120.4
C14—O3—K^iii^	129.98 (12)	C20—C21—C16	121.71 (18)
C14—O4—H1O	106.2	C20—C21—H21	119.1
C15—O5—K	128.86 (13)	C16—C21—H21	119.1
C15—O6—K^ii^	143.84 (11)	C27—C22—C23	118.46 (18)
C15—O6—H2O	109.1	C27—C22—S4	121.72 (15)
K^ii^—O6—H2O	80.5 (18)	C23—C22—S4	119.78 (15)
C28—O7—K^iii^	138.62 (13)	C24—C23—C22	119.70 (18)
C28—O8—H3O	108.8	C24—C23—C28	119.61 (17)
O1—C1—O2	123.42 (17)	C22—C23—C28	120.65 (18)
O1—C1—C2	118.14 (17)	C25—C24—C23	121.10 (19)
O2—C1—C2	118.43 (15)	C25—C24—H24	119.5
O1—C1—K	50.75 (9)	C23—C24—H24	119.5
O2—C1—K	89.28 (10)	C24—C25—C26	118.9 (2)
C2—C1—K	132.00 (11)	C24—C25—H25	120.5
C7—C2—C3	119.20 (17)	C26—C25—H25	120.5
C7—C2—C1	116.45 (16)	C27—C26—C25	121.0 (2)
C3—C2—C1	124.35 (16)	C27—C26—H26	119.5
C4—C3—C2	118.82 (17)	C25—C26—H26	119.5
C4—C3—S1	121.17 (14)	C26—C27—C22	120.77 (19)
C2—C3—S1	120.00 (14)	C26—C27—H27	119.6
C5—C4—C3	120.82 (18)	C22—C27—H27	119.6
C5—C4—H4	119.6	O7—C28—O8	123.05 (18)
C3—C4—H4	119.6	O7—C28—C23	122.69 (18)
C4—C5—C6	120.47 (19)	O8—C28—C23	114.24 (17)
C4—C5—H5	119.8		
			
C3—S1—S2—C8	99.20 (9)	C5—C6—C7—C2	−0.1 (3)
K^ii^—S1—S2—C8	−127.65 (6)	C3—C2—C7—C6	−0.1 (3)
C17—S3—S4—C22	−80.05 (9)	C1—C2—C7—C6	179.66 (18)
K^ii^—S3—S4—C22	140.98 (7)	S1—S2—C8—C13	−14.36 (17)
O7^i^—K—O1—C1	−105.72 (13)	S1—S2—C8—C9	162.90 (14)
O3^i^—K—O1—C1	171.05 (13)	C13—C8—C9—C10	4.5 (3)
O5—K—O1—C1	−49.30 (12)	S2—C8—C9—C10	−172.87 (14)
O2^ii^—K—O1—C1	15.19 (13)	C13—C8—C9—C14	−173.11 (17)
O6^ii^—K—O1—C1	69.87 (12)	S2—C8—C9—C14	9.5 (2)
S1^ii^—K—O1—C1	53.30 (16)	C8—C9—C10—C11	−1.9 (3)
S3^ii^—K—O1—C1	109.82 (12)	C14—C9—C10—C11	175.66 (17)
O7^i^—K—O5—C15	−153.39 (17)	C9—C10—C11—C12	−1.7 (3)
O3^i^—K—O5—C15	123.90 (16)	C10—C11—C12—C13	2.8 (3)
O1—K—O5—C15	68.98 (16)	C11—C12—C13—C8	−0.2 (3)
O2^ii^—K—O5—C15	−41.24 (16)	C9—C8—C13—C12	−3.5 (3)
O6^ii^—K—O5—C15	11.17 (17)	S2—C8—C13—C12	173.84 (15)
S1^ii^—K—O5—C15	−83.26 (16)	K^iii^—O3—C14—O4	29.1 (3)
C1—K—O5—C15	52.17 (16)	K^iii^—O3—C14—C9	−152.68 (13)
S3^ii^—K—O5—C15	−3.0 (2)	C10—C9—C14—O3	−179.17 (18)
K—O1—C1—O2	−56.0 (2)	C8—C9—C14—O3	−1.6 (3)
K—O1—C1—C2	122.87 (14)	C10—C9—C14—O4	−0.8 (2)
K^ii^—O2—C1—O1	141.59 (18)	C8—C9—C14—O4	176.81 (17)
K^ii^—O2—C1—C2	−37.3 (3)	K—O5—C15—O6	30.2 (3)
K^ii^—O2—C1—K	101.6 (2)	K—O5—C15—C16	−149.79 (13)
O7^i^—K—C1—O1	92.45 (13)	K^ii^—O6—C15—O5	−96.3 (2)
O3^i^—K—C1—O1	−8.48 (12)	K^ii^—O6—C15—C16	83.7 (2)
O5—K—C1—O1	126.07 (13)	O5—C15—C16—C21	−4.0 (3)
O2^ii^—K—C1—O1	−165.27 (12)	O6—C15—C16—C21	176.03 (17)
O6^ii^—K—C1—O1	−99.29 (13)	O5—C15—C16—C17	175.73 (18)
S1^ii^—K—C1—O1	−147.01 (11)	O6—C15—C16—C17	−4.2 (3)
S3^ii^—K—C1—O1	−72.07 (12)	C21—C16—C17—C18	0.1 (3)
O7^i^—K—C1—O2	−131.37 (10)	C15—C16—C17—C18	−179.68 (17)
O3^i^—K—C1—O2	127.70 (10)	C21—C16—C17—S3	−178.20 (14)
O1—K—C1—O2	136.18 (18)	C15—C16—C17—S3	2.1 (3)
O5—K—C1—O2	−97.75 (11)	S4—S3—C17—C18	11.76 (17)
O2^ii^—K—C1—O2	−29.09 (12)	K^ii^—S3—C17—C18	147.07 (13)
O6^ii^—K—C1—O2	36.89 (9)	S4—S3—C17—C16	−170.00 (14)
S1^ii^—K—C1—O2	−10.83 (13)	K^ii^—S3—C17—C16	−34.69 (18)
S3^ii^—K—C1—O2	64.11 (10)	C16—C17—C18—C19	−0.1 (3)
O7^i^—K—C1—C2	−2.37 (18)	S3—C17—C18—C19	178.21 (16)
O3^i^—K—C1—C2	−103.30 (15)	C17—C18—C19—C20	−0.3 (3)
O1—K—C1—C2	−94.8 (2)	C18—C19—C20—C21	0.6 (3)
O5—K—C1—C2	31.25 (15)	C19—C20—C21—C16	−0.6 (3)
O2^ii^—K—C1—C2	99.91 (15)	C17—C16—C21—C20	0.3 (3)
O6^ii^—K—C1—C2	165.89 (17)	C15—C16—C21—C20	−179.95 (18)
S1^ii^—K—C1—C2	118.17 (15)	S3—S4—C22—C27	−3.69 (18)
S3^ii^—K—C1—C2	−166.89 (15)	S3—S4—C22—C23	173.96 (14)
O1—C1—C2—C7	−19.6 (2)	C27—C22—C23—C24	1.9 (3)
O2—C1—C2—C7	159.39 (17)	S4—C22—C23—C24	−175.88 (15)
K—C1—C2—C7	41.5 (2)	C27—C22—C23—C28	−175.62 (18)
O1—C1—C2—C3	160.18 (18)	S4—C22—C23—C28	6.6 (3)
O2—C1—C2—C3	−20.8 (3)	C22—C23—C24—C25	−1.3 (3)
K—C1—C2—C3	−138.76 (15)	C28—C23—C24—C25	176.20 (19)
C7—C2—C3—C4	0.6 (3)	C23—C24—C25—C26	−0.7 (3)
C1—C2—C3—C4	−179.18 (18)	C24—C25—C26—C27	2.1 (3)
C7—C2—C3—S1	−179.68 (14)	C25—C26—C27—C22	−1.5 (3)
C1—C2—C3—S1	0.6 (3)	C23—C22—C27—C26	−0.5 (3)
S2—S1—C3—C4	0.94 (18)	S4—C22—C27—C26	177.21 (16)
K^ii^—S1—C3—C4	−132.26 (15)	K^iii^—O7—C28—O8	−32.8 (3)
S2—S1—C3—C2	−178.79 (13)	K^iii^—O7—C28—C23	145.73 (16)
K^ii^—S1—C3—C2	48.01 (17)	C24—C23—C28—O7	−171.4 (2)
C2—C3—C4—C5	−0.8 (3)	C22—C23—C28—O7	6.1 (3)
S1—C3—C4—C5	179.44 (17)	C24—C23—C28—O8	7.2 (3)
C3—C4—C5—C6	0.6 (4)	C22—C23—C28—O8	−175.31 (18)
C4—C5—C6—C7	−0.1 (4)		

Symmetry codes: (i) *x*, *y*+1, *z*; (ii) −*x*, −*y*+1, −*z*+1; (iii) *x*, *y*−1, *z*.

### Hydrogen-bond geometry (Å, °)

**Table:**

*D*—H···*A*	*D*—H	H···*A*	*D*···*A*	*D*—H···*A*
O4—H1o···O1^iii^	0.84	1.80	2.631 (2)	169
O6—H2o···O2^ii^	0.84	1.68	2.515 (2)	177
O8—H3o···O5^iii^	0.84	1.88	2.704 (2)	167

Symmetry codes: (iii) *x*, *y*−1, *z*; (ii) −*x*, −*y*+1, −*z*+1.
